# Hepatitis E Virus Epidemiology in Industrialized Countries

**DOI:** 10.3201/eid0904.020351

**Published:** 2003-04

**Authors:** Pilar Clemente-Casares, Sonia Pina, Maria Buti, Rosend Jardi, Margarita Martín, Sílvia Bofill-Mas, Rosina Girones

**Affiliations:** * University of Barcelona, Barcelona, Spain; † Hospital General Universitario Valle Hebron, Barcelona, Spain; ‡ Universitat Autònoma de Barcelona, Barcelona, Spain

**Keywords:** Acute hepatitis, HEV, swine HEV, environmental strains, urban sewage, HAV, research

## Abstract

To determine the prevalence of Hepatitis E virus (HEV) in industrialized nations, we analyzed the excretion of HEV strains by the populations of Spain, France, Greece, Sweden, and the United States. Twenty of 46 (43.5%) urban sewage samples collected in Barcelona from 1994 to 2002 tested positive for HEV. We identified 15 HEV strains, which were similar to two HEV isolates previously described in Barcelona in clinical samples and to strains from diverse geographic HEV-nonendemic areas. We also identified two HEV strains in sewage samples from Washington, D.C., and Nancy, France; these samples were also positive for Hepatitis A virus. In addition, we studied the role of pigs as a reservoir for HEV and identified one new swine HEV strain. Our results suggest that HEV may be more prevalent than previously considered in industrialized countries and that variants of the virus circulate simultaneously in one region.

Hepatitis E virus (HEV) infection is a major cause of epidemic and acute sporadic hepatitis in many areas of Asia, Africa, and Mexico (*1*,*2*), where HEV is considered endemic. HEV is an RNA virus enterically transmitted with a single serotype, which affects mainly young adults. In countries where the virus is endemic, HEV is associated with >50% of sporadic acute hepatitis cases. The disease is self-limited but sometimes has severe complications and a high case-fatality rate, particularly in pregnant women (approximately 20%) (*3*). North America and Europe have traditionally been considered nonendemic for HEV; most HEV infections in those regions are considered to be imported, although seroprevalence ranges from 1% to 5% (*4*). In the last few years, some HEV strains associated with sporadic acute hepatitis have been isolated from human serum samples in North America (*5*) and Europe (i.e., Italy, Greece, Spain, and the United Kingdom) (*6*,*7*). Molecular analyses have shown that these strains form a group of HEV isolates that are genetically divergent compared with strains from HEV-endemic countries (*8*).

Evidence also exists that some animals can be reservoirs of HEV; for example, HEV infection has been demonstrated in swine (*9*). Swine and human HEV strains from a particular geographic region often appear to be closely related genetically (*6*,*9*–*11*). Transmission of HEV infection during outbreaks primarily occurs through contaminated water (*12*). Unlike other enterically transmitted infections, person-to-person transmission of HEV occurs infrequently (*13*).

We investigated the level of infection in regions where HEV is considered nonendemic by analyzing the excreted virus in the urban sewage of diverse geographic areas. The excretion and epidemiology of HEV was compared to the excretion and epidemiology of Hepatitis A virus (HAV) (*14*). We also analyzed swine fecal samples to identify evidence of HEV infection in pigs.

## Material and Methods

### Sewage Samples

Urban sewage samples were collected at the entry of a water treatment plant receiving 670,000 m^3^/day of waste products from Barcelona, Spain (population: approximately 1.8 million inhabitants). From the sewage network of this city, we collected 26 samples (taken every 2 weeks) from June 2000 to May 2001 and 8 samples (taken monthly) from June 2001 to January 2002. We also tested 12 sewage samples previously collected from the same area and stored at –80°C: 2 from 1994, 2 from 1995, 2 from 1996, 3 from 1997, 1 from 1998, and 2 from 1999. Each sample was collected in a sterile 500-mL polyethylene container, kept at 4°C for <8 h until viral particles were concentrated in phosphate-buffered saline (PBS, pH 7.3), and stored at –80°C.

We obtained urban sewage samples from other countries, as follows: five samples from Patras, Greece, in June–July 1999; five samples from Washington, D.C., United States, in December 1999; four samples from Nancy, France, in March 1998; and four samples from Umeå, Sweden, in September–October 1997. These samples were collected and shipped, frozen, to Spain where the viral particles were concentrated in PBS and stored at –80°C.

### Human Serum Samples

We contacted 13 patients seen in the emergency room of the Hospital General Valle Hebron, Barcelona, Spain, for acute hepatitis over the last 12 years who had tested positive for immunoglobulin (Ig) G anti-HEV. A follow-up serum sample was collected in order to evaluate the durability of antibody response. These samples were stored at –80°C.

### Animal Samples

Seventy-three serum samples were obtained from healthy pigs in three commercial herds in Catalonia, Spain: 43 weaned pigs (3–9 weeks old), 10 fattening pigs (10–22 weeks old), 8 gilts (young sows), and 12 sows. Blood samples were taken aseptically from the vena cava or jugular vein, and the serum obtained by centrifugation was stored at –80°C until tested.

Thirty fecal samples from pigs of various ages were collected from the same three herds. Individual samples were taken and later pooled with samples from 3–5 animals in the same age group. Samples from the “fattening” pigs were collected from different places in their pens, each one containing 10–15 animals.

### Serologic Tests of Human Serum Samples

Commercially available enzyme immunoassays (Abbott Laboratories, Abbott Park, IL) were used for the detection of hepatitis B surface antigen, IgM anti–hepatitis B core, IgM anti-hepatitis C virus (HCV), anti-HAV, and IgG anti-HEV. HCV-RNA was analyzed by polymerase chain reaction (PCR) (Amplicor HCV, monitor test version 2.0; Hoffman-La Roche Inc., Nutley, NJ).

### Serologic Tests of Swine Serum Samples

A commercially available enzyme immunoassay (Abbott Laboratories) was used, with some modifications to detect IgG anti-HEV in pigs. Hyperimmune serum from a pig experimentally inoculated with the porcine-HEV strain and preimmune serum from a noninoculated pig and a Rhesus Monkey (*Macaca mulatta*) (serum donated by S.U. Emerson and R.H. Purcell) were used as positive and negative controls, respectively.

### Control Viruses

Fecal suspensions obtained from Rhesus Monkeys infected with HEV Barcelona (BCN) strain (10% in PBS, pH 7.3) were used as positive control for the PCR analysis. The strain that infected the monkeys was isolated from sewage in Barcelona and is genetically similar to Indian strains (*15*). Viral suspensions were stored at –80°C.

### Concentration of Viral Particles from Sewage Samples

Recovery of viral particles was carried out as described previously (*16*,*17*). Briefly, a 40-mL sewage sample was ultracentrifuged (110,000 × *g* for 1 h at 4°C) to pellet all viral particles together with any suspended material. The sediment was eluted by using 4 mL 0.25 N glycine buffer, pH 9.5, and the suspended solids were separated by centrifugation at 12,000 × *g* for 15 min. Viruses were finally pelleted by using ultracentrifugation (110,000 × *g* for 1 h at 4°C), resuspended in 0.1 mL PBS, and stored at –80°C.

### Concentration of Viral Particles from Swine Feces Samples

A protocol similar to the one used for sewage samples was carried out to concentrate viral particles from swine feces samples; 1 g of a pool of feces was eluted in 4 mL 0.25 N glycine buffer, pH 9.5, and centrifuged (10,000 × *g* for 15 min) to separate suspended solids. The supernatant was finally ultracentrifuged (110,000 × *g* for 1 h at 4°C), and viruses were resuspended in 0.1 mL PBS and stored at –80°C.

### Nucleic Acid Extraction

Viral nucleic acids from viral particles were extracted as described previously (*18*) after we selected the procedure that is more likely to eliminate potential inhibitors of reverse transcription (RT)–PCR. The method is based on the use of guanidinium isothiocyanate and adsorption of the nucleic acids to silica particles. Briefly, 50 µL of viral concentrate was added to a mixture of 50 µL of silica particle suspension and 900 µL of lysis buffer. The mixture was incubated at room temperature for 10 min and washed twice in 1 mL of washing buffer (12 g of guanidine thiocyanate in 10 mL of Tris-EDTA), twice more in 70% ethanol, and once in acetone. The pellet obtained after the complete evaporation of acetone was resuspended with 50 µL of elution buffer (49.4 µL of dithiothreitol [DTT] and 0.6 µL of RNase inhibitor [Applied Biosystems, Foster City, CA]). The extracted nucleic acids were then used for cDNA synthesis and amplification of the HEV and HAV genomes.

### Enzymatic Amplification

To detect viral RNA, we used a seminested RT-PCR with degenerated primers as described (*19*). Five µL of the extracted nucleic acids and a 10-fold dilution (corresponding to 5 µL and 0.5 µL of serum, 50 mg and 5 mg of feces, and 2 mL and 0.2 mL of sewage) were analyzed by RT-PCR, plus 1.5 mM of MgCl_2_, PCR Gold Buffer (10 mM Tris-HCl, pH 8.3, 50 mM KCl) (Applied Biosystems), 0.01 M DTT, 10 nmol of each dNTP, and 25 pmol of the reverse primer (HEVORF2con-a1; nt 6454–6479 in HEV United States [US1] strain) in a total volume of 10 µL. The mixture was incubated at 95°C for 5 min before the addition of 10 U of ribonuclease inhibitor (Applied Biosystems) and 50 U of RT mouse mammary leukemia virus (Applied Biosystems). After 30 min at 42°C, the mixture was heated again at 95°C for 5 min.

For a typical one-step reaction, we used 10 µL of the cDNA solution. Amplification was carried out in a 50-µL reaction mixture containing PCR Gold Buffer (Applied Biosystems), 1.2 mM MgCl_2_, 2 U of Ampli Taq Gold (Applied Biosystems) 25 pmol of the forward primer (HEVORF2con-s1; nt 6283–6306 in the HEV US1 strain). The first cycle of denaturation was carried out for 5 min at 95°C, followed by 35 cycles at 94°C for 60 s, annealing at 55°C for 30 s, and extension at 72°C for 1 min. All amplifications were completed with a 10-min, 72°C extension period. For a second PCR amplification cycle, we added 1 µL of the first-round product to a new batch of 50-µL reaction mixture containing 25 pmol of each primer (HEVORF2con-a1 and HEVORF2con-s2; nt 6332–6353 in US1 strain). This second PCR was performed under the same conditions. The products were analyzed by agarose gel electrophoresis with ethidium bromide stain. A region of 148 nucleotides within open reading frame (ORF) 2 of the HEV genome was amplified. Also, assays for the amplification of a region of 287 nucleotides within ORF1 were carried out. Primers used were HEVORF1con-s1 (nt 2–25), HEVORF1con-s2 (nt 50–70), HEVORF1con-a1 (nt 397–419), and HEVORF1con-a2 (nt 313–336). The primer positions are relative to HEV US1 strain. Nested RT-PCR was carried out for detecting HAV genome in sewage samples as described (*14*), amplifying fragments within the 5´ nontranslated region (NTR) region and the VP1/A2 junction region.

### Quality Control of the Amplification Method

Standard precautions were used for all procedures to reduce the possibility of sample contamination by amplified DNA molecules. A negative control was added every two samples. Virus-positive amplification results were confirmed by sequencing.

### Sequencing and Analysis of the Viral Genome

The amplicons obtained after the nested PCR were purified by using the QIAquick PCR purification Kit (QIAGEN GmbH, Inc., Hilden, Germany), following the manufacturer’s instructions. Both strands of the purified DNA amplicons were sequenced with the ABI PRISM BigDye Terminator Cycle Sequencing Ready Reaction kit with Ampli Taq DNA polymerase FS (Applied Biosystems), following the manufacturer’s instructions. We checked results using the ABI PRISM 3700 DNA analyzer (Applied Biosystems). We compared the sequences with those present in GenBank and the European Molecular Biology Library by using the basic BLAST program of the National Center for Biotechnology Information (available from: URL: http://www.ncbi.nlm.nih.gov/BLAST/). GenBank accession numbers of the HEV strains sequences characterized previously and used for phylogenetic studies are listed in Table 1.

**Table 1:** Nucleotide sequence accession numbers for Hepatitis E virus strains

Origin	Abbreviation	GenBank accession no.
Described in previous studies		
Barcelona	BCN	AF058684
Greece	G1	AF110391
	G2	AF110392
Italy	It	AF110390
Austria	Au	AF279123
United States	US1	AF060668
	US2	AF060669
	Sw^a^	AF082843
Mexico	M	M74506
Pakistan	P	M80581
Burma	B	M73218
China	C1	D11092
	C2	AJ272108
	C3	AF082094
India	I	X98292
Reported in this study		
Barcelona	BCN2	AF491004
	BCN3	AF490985
	BCN4	AF491003
	BCN5–BCN16	AF490986–AF490997
Washington, D.C., United States	W1	AF490998
Nancy, France	N1	AF490999
Barcelona, Spain^b^	VH1	AF491000
	VH2	AF491001
Catalonia, Spain^a^	Por1	AF491002

^a^Originated from swine. ^b^Strains reported previously (15), but their sequences within open reading frame 2 used for phylogenetic analysis in this study are new.

Alignments of the sequences were carried out by using the ClustalX 1.8 program (available from: ftp://ftp-igbmc.u-strasbg.fr/pub/ClustalX/). Phylograms were generated by the UPGMA algorithm using the NEIGHBOR program. The robustness of the grouping was determined by bootstrap resampling of the multiple sequence alignments (1,000 sets) with the programs SEQBOOT, DNADIST, NEIGHBOR, and CONSENSE. The output graphics of the trees were created with the TREEVIEW package, version 1.5 (*20*).

## Results

### Sewage Samples

Forty-six sewage samples from Barcelona were collected from 1994 to 2002. Of the samples collected before 2001, a total of 4 (14.9%) of 27 tested positive for HEV RNA; this proportion increased to 84.2% (16/19) among the samples collected from 2001 to 2002 (Table 2). After performing sequence analysis of the amplified region within ORF2, we identified 15 new HEV isolates (BCN2–BCN16) (GenBank accession nos. in Table 1).

**Table 2:** Summary of results of HEV and HAV found in urban sewage samples^a^

Site	Period of sampling	Positive HEV samples/total analyzed	Positive HAV samples/total analyzed
Barcelona, Spain	October–November 1994	0/2	1/2
	May–June 1995	0/2	1/2
	February–April 1996	1/2	1/2
	September–October 1997	0/3	2/3
	January 1998	0/1	1/1
	March–April 1999	1/2	2/2
	June–December 2000	2/15	13/15
	January 2001–January 2002	16/19	NT
Total (for Barcelona)		20/46 (43.5%)^b^	21/27 (77.8%)^b^
Nancy, France	March 1998	1/4	3/4
Umeå, Sweden	September–October 1997	0/4	1/4
Patras, Greece	June–July 1999	0/5	1/4
Washington, D.C., United States	December 1999	1/5	5/5

^a^HEV, Hepatitis E virus; HAV, Hepatitis A virus; NT, not tested. ^b^% of positive samples.

HAV RNA was detected in 21 (77.8%) of 27 sewage samples that were collected in Barcelona from 1994 to 2000 and tested for the presence of HAV. Previous studies in the same area showed 31 (57.4%) of 54 sewage samples positive for HAV from 1994 to 2000 (*14*).

We also tested for the presence of HEV RNA in sewage samples from other countries where HEV was previously considered nonendemic. One of five samples from Washington, D.C., and one of four samples tested from Nancy, France, were positive, showing two new HEV strains (W1 and N1). HEV RNA was not detected in any of the four samples from Umeå, Sweden, or the five samples from Patras, Greece. HAV RNA was detected in all four countries: All U.S. sewage samples, three of four samples from France, one of five samples from Greece, and one of four from Sweden tested positive for HAV.

### Human Serum Samples

Follow-up serum samples from 13 seropositive acute hepatitis patients from Barcelona were analyzed to evaluate the durability of antibody response. None of these samples showed the RNA of the virus. IgG anti-HEV was undetectable in 7 (53.8%) of 13 of these follow-up samples. Seven of eight seropositive patients were found to be seronegative 2 to 12 years after the initial test.

### Animal Samples

Swine serum and fecal samples from pigs in three different herds in Catalonia, Spain, were tested. A total of 10 (13.7%) of 73 serum samples were positive for anti-HEV IgG antibodies (all were obtained from fattening pigs or the oldest pigs from the same herd). A total of 55 serum samples from pigs of different ages were taken at this farm. The distribution of seropositive animals was as follows: 4 (40.0%) of 10 samples from fattening pigs, 2 (40.0%) of 5 from gilts, 1 (33.3%) of 3 from primiparous sows, and 3 (42.8%) of 7 from multiparous sows. Pigs from the other two farms were seronegative for HEV. None of the serum samples were positive for HEV by RT-PCR.

The HEV genome was amplified by RT-PCR in 6 of 12 fecal samples collected in the herd with seropositive animals. We detected HEV in one of the two pools from pigs 3 weeks old, in one of two pools from pigs 5 weeks old, in two of two pools from pigs 8 weeks old, in one of two pools from fattening pigs, and in the pool of feces from primiparous sows. After sequence analysis, we identified one new swine HEV strain (Por1).

### Sequence Analysis of the HEV RNA Genomes

**Figure 1:**
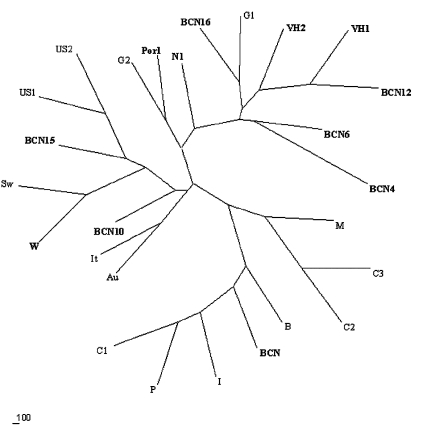
Unrooted phylogenetic tree showing the relationship over a 123-bp fragment within open reading frame 2 between representative Hepatitis E virus strains reported in this study and other isolates from genotype I (C1, China; P, Pakistan; I, India; BCN, Barcelona, Spain; and B, Burma), genotype II (M, Mexico), genotype III (US1 and US2, United States; Sw, swine; G1 and G2, Greece; It, Italy; and Au, Austria), and genotype IV (C2 and C3, China). Strains from Barcelona, Spain, Washington, D.C., United States, and Nancy, France, are in black.

**Figure 2:**
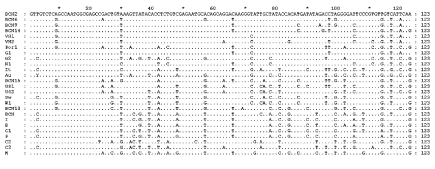
Nucleotide alignment of the amplified fragment within ORF2 from some representative isolates in this study with other Hepatitis E virus strains. Dots indicate sequence identities.

Phylogenetic analysis showed that all the sequences detected were grouped with strains isolated in countries where HEV was considered nonendemic (Figure 1). The nucleotide sequence alignment of the detected strains with other HEV strains is shown in Figure 2. BCN2–9, BCN11–BCN14, and BCN16 shared a 91.0% to 99.2% similarity. They were closely related to VH1 (94.3% to 98.4% identity) and VH2 (91.0% to 95.1%), two strains isolated from clinical samples in the same area (Barcelona), previously described (*15*), and G1 (92.7% to 96.7%), an isolate from Greece. Two isolates (BCN10 and BCN15) showed substantially different sequences, sharing 86.5% to 89.2% and 87.2% to 91.9% similarity with the rest of strains detected in Barcelona. A previous study (*15*) in the same area with specific primers for HEV strains from areas where the virus is endemic identified one isolate (BCN), which was closely related to Indian strains. Nucleotide sequence identities among this isolate and the new ones from Barcelona ranged from 79.7% to 83.7%.

The HEV strain identified in sewage from Washington (W1) was closely related to other isolates from the United States, sharing 91.0% to 91.9% identity with US1 and US2 strains obtained from human serum samples, and 98.4% with a swine strain (Sw) also isolated in the United States. BCN15, from Barcelona, was also similar to those isolates from the United States, showing 89.4% to 91.9% nucleotide sequence identity. The sample from Nancy (N1) was highly similar (92.7%) to VH1. All HAV strains were genotype IA, except the Swedish isolate (IB).

**Figure 3:**
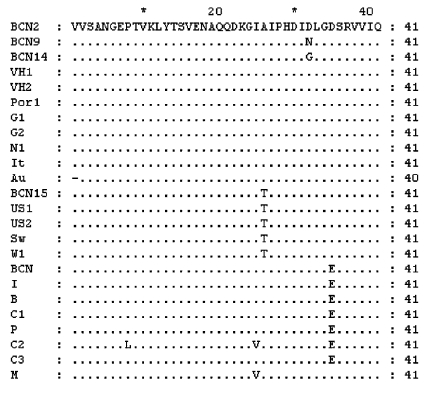
Amino acid alignment from representative isolates in this study with other Hepatitis E virus strains. Amino acid sequences from BCN3–BCN8, BCN10–BCN13, and BCN16 are identical to BCN2. Dots indicate sequence identities.

The nucleotide sequences of the isolates from swine were identical and, when compared with other isolates, the Por1 strain exhibited a similarity of 92.7% with G2 and 87.0% to 91.0% with isolates from sewage in Barcelona. The results of translating nucleotide sequences into amino acid sequences showed that the substitutions in strains detected in Barcelona (including the one from pigs) were mainly located in the third codon position and were conservative (Figure 3). Two strains (BCN9 and BCN14) showed two nonconservative substitutions in the same position.

## Discussion

In previous studies, investigating the viruses present in a population’s sewage has produced reliable information about the strains infecting that population; this type of analysis detects the viruses that cause clinical and subclinical infections in the population (*14*). In our study, we showed the presence of HEV strains during a period of several years in raw urban sewage from an area previously considered nonendemic for the virus. We also demonstrated the presence of HEV in other industrialized countries by testing urban sewage samples from diverse geographic areas. We identified 17 new HEV strains from sewage samples in Barcelona, Spain (15 isolates); Washington, D.C., United States (1 isolate); and Nancy, France (1 isolate); these locations also produced a high percentage of HAV-positive sewage samples.

The amplified region of the viral genomes detected in this study was sequenced; the diversity we observed indicates the absence of a unique HEV outbreak in the population and shows that a diverse number of strains are simultaneously circulating. Only the viral genomes from the samples showing the higher viral concentration (Por1 and W1) could be sequenced in ORF1 by using the described primers. Y. Wang et al. (*21*) noted that negative results were observed in serum samples positive in the ORF2 when using these ORF1 primers, suggesting a lower level of efficiency in the amplification reactions compared with the ORF2 primers applied in the study. The phylogenetic analysis of short sequences has previously produced trees of more similar structure than those observed with longer sequences (*21*,*22*). The strains we describe may be most closely related to genotype III, one of the four genotypes described by G.G. Schlauder and I.K. Mushahwar (*8*). Larger fragments should be sequenced in order to confirm these results.

The prevalence of HAV infection in Catalonia, where most of the sewage samples were collected, is 67.8% (*23*); in this area, HAV is considered to be of intermediate endemicity. In the same area of Barcelona, 57.4% of the sewage samples collected from 1994 to 2000 showed HAV; these HAV strains were found to be closely related to the HAV strains identified in the clinical samples for the same period (*14*).

HEV can be detected in the stool of most patients infected with HEV, with duration of fecal shedding generally limited to 9–12 days (*24*). If we compare the transmission of HEV and HAV in this region, since the time after infection that HEV is excreted does not appear to be longer than the time after infection that HAV is excreted, we find that HEV infections may be more frequent than previously considered. That 84.2% of sewage samples collected during 2001 and 2002 were HEV positive suggests that the prevalence of HEV infections may be underestimated.

In our study we also analyzed samples from three commercial pig farms; only one of these farms had pigs positive for HEV. However, from that one farm, antibodies and virus were detected in animals of different ages, suggesting that HEV is widespread among animals in infected herds. Our serologic results showed that although only 13.7% of samples were positive, all of them were from animals >10 weeks (fattening pigs and sows). Since that farm worked with a farrow-to-finish system, these results may indicate a slow diffusion of infection within the farm. The fact that adult sows had positive serologic and virologic test results suggests their possible role as reservoirs on the farm. The swine HEV strain identified in these animals has been found to be related to the human strains detected (87.0% to 91.0%). Previous studies also reported that swine HEV isolates identified in the United States (*9*), Taiwan (*11*), and Japan (*10*) were closely related to human HEV isolates obtained from the same geographic areas. In fact, swine HEV isolates seem to be more similar to human HEV isolates from the same area than they are to swine HEV isolates from different geographic areas (*25*). Swine HEV isolates identified in Canada and the Netherlands have been clustered with previously described European and American human or swine isolates (*26*,*27*). Finally, veterinarians and persons working with pigs have been shown to at a greater risk for HEV infection. Meng et al. (*28*) reported that swine veterinarians were one and a half times more likely to seroconvert to HEV infection, although clearly multiple sources of exposure can exist.

Most existing assays for antibody to HEV are enzyme immunoassays that use recombinant-expressed proteins or synthetic peptides representing antigenic domains from ORF 2 and ORF 3, commonly from strains of at least two geographically distinct HEV strains. The HEV strains used in these tests are representative of those from countries with endemic HEV and show some differences from recently identified strains in the sequence of amino acids of several major epitopes as the region near the carboxyl ends of ORF 2 and ORF 3. Some differences are shown in amino acid sequences of strains from areas with nonendemic and endemic virus in the amplified region, which is located within a strongly reactive epitope (*29*). This diversity could be producing a lower level of sensitivity in the serologic assays for these infections. A recent study from the Hepatitis E Virus Antibody Serum Panel Evaluation Group concluded that discrepant results among blood donor serum samples show that anti-HEV seroprevalence data in countries with nonendemic HEV may be unreliable and should be interpreted with caution (*30*). Our results also support this conclusion. As some authors have suggested (*31*), we show that IgG antibodies a few years after the initial test are reduced to undetectable levels in 53.8% of cases. In some cases, antibody levels are reduced very early, after only 3–4 months, which makes the diagnosis of HEV infection extremely difficult. Several authors have suggested that some persons may not produce a detectable antibody response at all (*24*). Balayan (*3*) also considered the prevalence of anti-HEV in areas endemic for HEV to be much lower than expected, with a rate of 2.8% to 20.2% in areas having a high proportion of HAV-seropositive persons. In highly industrialized countries, anti-HEV has been regularly found at a rate not exceeding 5%; in Spain, anti-HEV was found in 1% to 3% of blood donors (*32*).

Specific information on the pathogenicity of the HEV strains detected in Europe and the United States is not available; these data are required for evaluating the potential risk associated with the infection. Using adequate specific tests must be considered for the routine diagnosis of HEV infections in patients with acute viral hepatitis not related to HAV in industrialized countries, especially in asymptomatic patients with unexplained elevated aminotransferase levels and in pregnant women. Our data suggest that HEV strains are more widespread in the human population than previously thought and endemic HEV infections are likely present in Europe and the United States. More studies are warranted to characterize the HEV infections detected and evaluate the sanitary risk that the excreted HEV represents for humans.
